# Imputation of missing clock times – application to procalcitonin concentration time course after birth

**DOI:** 10.1007/s10928-025-09965-8

**Published:** 2025-03-18

**Authors:** Abigail J. Bokor, Nick Holford, Jacqueline A. Hannam

**Affiliations:** Department of Pharmacology and Clinical Pharmacology, The University of Auckland, 85 Park Road, Grafton, Auckland, 1023 New Zealand

**Keywords:** Imputation, Non-linear mixed effect model, Procalcitonin, Neonate, Birth

## Abstract

The time course of biomarkers (e.g., acute phase proteins) are typically described using days relative to events of interest, such as surgery or birth, without specifying the sample time. This limits their use as they may change rapidly during a single day. We investigated strategies to impute missing clock times, using procalcitonin for population modelling as the motivating example. 1275 procalcitonin concentrations from 282 neonates were available with dates but not sample times (Scenario 0). Missing clock times were imputed using a random uniform distribution under three scenarios: (1) minimum sampling intervals (8–12 h); (2) procalcitonin concentrations increase for postnatal days 0–1 then decrease; (3) standard sampling practice at the study hospital. Unique datasets (*n* = 100) were created with scenario-specific imputed clock times. Procalcitonin was modelled for each scenario using the same non-linear mixed effects model using NONMEM. Scenarios were evaluated by the NONMEM objective function value compared to Scenario 0 (∆OFV) and with visual predictive checks. Scenario 3, based on standard sampling practice at the study hospital, was the best imputation procedure with an improved objective function value compared to Scenario 0 (∆OFV: -62.6). Scenario 3 showed a shorter lag time between the birth event and the procalcitonin concentration increase (average: 12.0 h, 95% interval: 9.7 to 14.3 h) compared to other scenarios (averages: 15.3 to 18.7 h). A methodology for selecting imputation strategies for clock times was developed. This may be applied to other problems where clock times are missing.

**Supplementary Information:**

The online version contains supplementary material available at 10.1007/s10928-025-09965-8.

## Introduction

Biomarkers can provide useful repeated observations for diagnosing and monitoring many clinical conditions, e.g. hypertension, anticoagulation and infection. However, their usefulness depends on the assumption that they are a good representation of the condition when the biomarker was observed. To describe the time course of a biomarker it is important to have accurately timed biomarker sampling with a sufficient number of samples [1, 2]. Studies investigating the concentration of acute phase proteins and cytokines following events such as birth [3, 4], burns [5, 6], surgery [7, 8] and trauma [9] typically provide concentration data using days instead of hours after an event of interest. Concentrations of these biomarkers may change within 8 h, with some increasing within 1.5 h [10, 11], so grouping the biomarker observations into a daily value discards information and may make interpretation challenging.

Procalcitonin is an acute phase protein for which concentrations are elevated during bacterial infection or sepsis [12, 13]. Procalcitonin increases within 4 h of an infection [14] and has been suggested as a useful tool in the diagnosis of infection, but concentrations are also increased after events such as birth [15] and surgery [7]. A dataset of procalcitonin concentrations following birth was explored using potential imputation scenarios to investigate the information loss arising from missing within day sample times. The objective of the study was to evaluate if the imputation of missing times could improve the description of the time course of procalcitonin concentrations by including the time of procalcitonin observations relative to hours, not days after birth. Scenarios based on expectations of clinical sampling procedures were investigated, not on specific characteristics of procalcitonin. This study describes a framework for imputing missing clock times that may be appropriate for other investigations where times are missing.

## Methods

An existing dataset of 282 Japanese neonates and infants born between 22 and 41 weeks gestational age was provided by the study investigators [16]. Serum procalcitonin (PCT) concentrations (*n* = 1275) were measured as part of routine patient care from birth up to 19 weeks postnatal age at the discretion of the attending physicians. Consequently, some patients contributed multiple observations, sometimes on the same day, and others only contributed one or two samples over several days. The dates of birth and procalcitonin observations were recorded, but the within day clock times of birth and procalcitonin sampling were not.

### Clock time scenarios for observations and birth event

Each event had a clock time imputed, hence the number of clock times is equal to the number of birth events (*n* = 282) plus the number of observations (*n* = 1275). Scenario 0 refers to the use of the original dataset without the imputation of clock times. A birth event was assumed to occur on the date of birth at a time of 00:00. All observations associated with a particular date were assumed to occur at 00:00 on that date, i.e. without imputation.

For other scenarios, clock times were imputed by randomly sampling from a uniform distribution (R Version 4.0.3; runif()). The imputed time for the birth event could be at any time point in the interval 00:00 to 23:59 (24 h clock), and procalcitonin observations on the day of birth could be collected at any time after this on the same day. For observations on subsequent days, the sample times were imputed between 00:00 to 23:59 on the day of the observation. Where multiple observations occurred on a single day, a minimum interval of 8 h between observations was defined because the process of sample collection, analysis and interpretation is timely and repeat samples are unlikely to be collected in close succession. This was undertaken by comparing the interval between observations to the minimum interval and resampling times for this day until this requirement was met. When there were too many observations on one day to be separated by an 8 h interval, the interval was reduced based on 24 divided by the number of samples on that day, rounded down to the nearest hour. For example, one patient had five samples collected in one day, so the minimum interval was reduced to four hours on that day. One hundred replicate datasets were generated for each of the three separate imputation scenarios which were considered the most clinically reasonable for sample collection as below:

1. Scenario 1: Multiple observations on the same day in the original dataset were assumed to be in the same sequence recorded by the investigators in the provided dataset. Minimum intervals between procalcitonin observations of 8 h (Scenario 1A) and 12 h (Scenario 1B) were investigated as part of this scenario.
2. Scenario 2: Multiple observations on the same day in the original dataset were assumed to increase and then decrease monotonically in time sequence. Based on the literature [15], procalcitonin concentrations were assumed to increase on postnatal days zero and one, then decrease on days two and three. Observations were reordered based on the procalcitonin concentrations within a day for each separate patient and times were imputed based on this new order.
3. Scenario 3: Standard clinical practice at the study hospital for taking blood samples was provided by the original study investigators. The first observation of each day was routinely collected at 9:00, with a minimum interval of 8 h between subsequent observations. The sequence of the observations provided in the original dataset was assumed to be the sequence in which they were observed. If the birth time was imputed at or after 09:00, the first observation on the day of birth was imputed after this time.

**Fig. 1:**
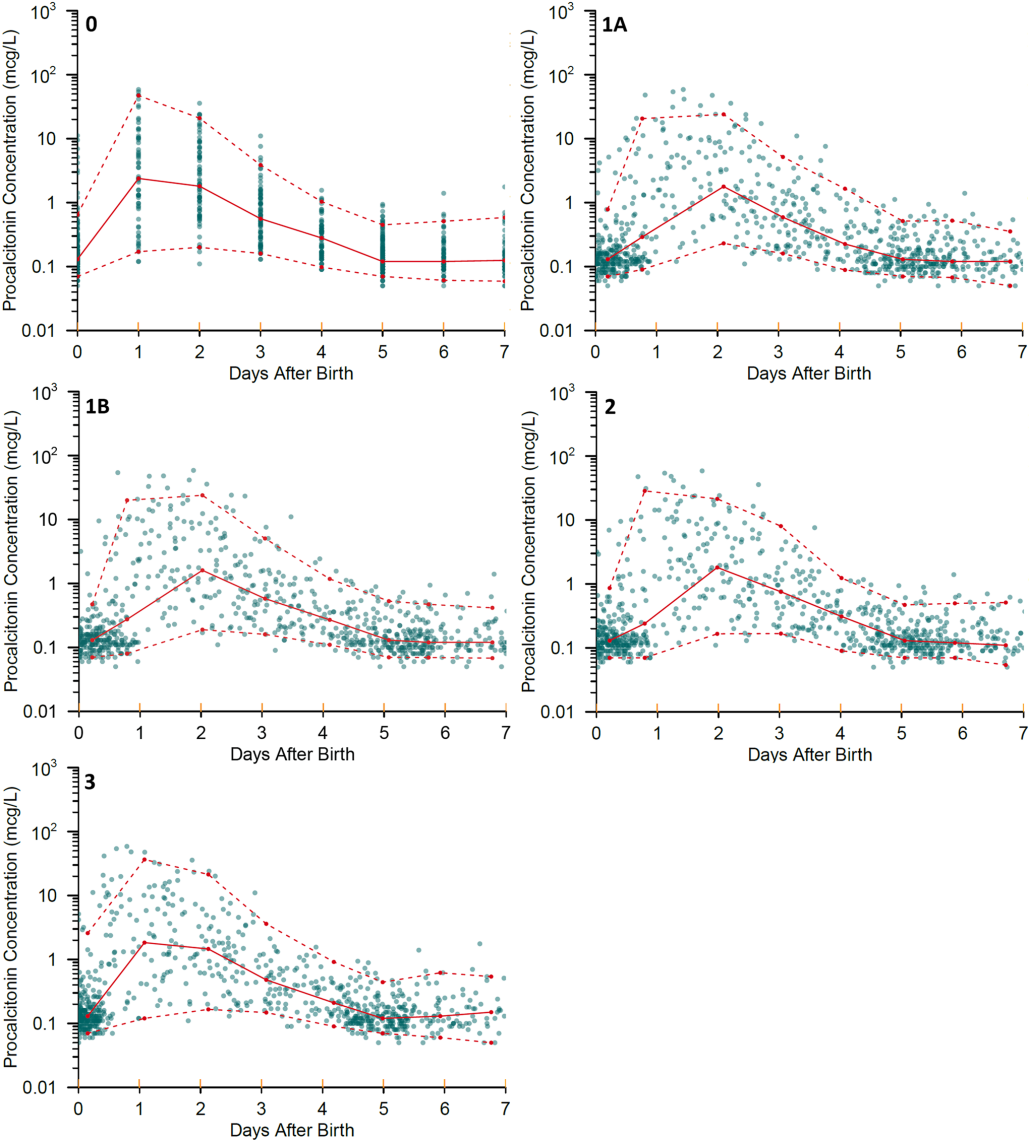
Observed procalcitonin concentrations (mcg/L) in the first seven postnatal days using sample times without imputation (Scenario 0), and with imputation: Scenario 1A, minimum interval of 8 h; Scenario 1B, minimum interval of 12 h; Scenario 2, peak at 1 day; Scenario 3, standard clinical practice at study hospital, 9am start. Coloured circles are the observed data points. Solid lines are medians of the observed values, whilst dashed lines are 5th and 95th percentiles of the observations

An example of the imputation of clock times for each scenario is shown in Fig. 1.

### Modelling methods

The model was developed based on the current data to allow comparison of imputation methods. A one compartment turnover model with first order elimination described by procalcitonin clearance (CL_PCT_) was used to describe the time course of procalcitonin concentration (C_PCT_) associated with the birth event (Eq. 1). The population volume of distribution of procalcitonin (V_PCT_) was assumed to be 15 L/70kg based on estimates of the volume of distribution of the cleaved product calcitonin [17]. The procalcitonin production rate (RateIn_PCT_) was assumed to be due to a stimulus (SYN_B_) associated with the birth event.

1 $$\:\frac{d{C}_{PCT}}{dt}=\:{RateIn}_{PCT}\:\times\:{SYN}_{B}-\:{C}_{PCT}\times\:{CL}_{PCT}$$

The time course of the birth effect stimulus was described by a one compartment model with a nominal bolus input of 1 unit to reflect the birth event and first order elimination with elimination half-life (Tel_B_). Tel_B_ is the half-life of the birth effect stimulus which describes the duration of the birth effect on the production rate of procalcitonin. A linear model linked the birth stimulus concentration (C_B_) to increased procalcitonin production using a slope parameter (SLOPE_B_), as shown in Eq. 2. A delay between the birth event and an increase in procalcitonin concentration was described using a lag time which delayed the onset of the birth effect relative to the time of the birth event.

2 $$\:{SYN}_{B}=1+({SLOPE}_{B}\times\:\:{C}_{B})$$

RateIn_PCT_ was used with CL_PCT_ to calculate baseline procalcitonin concentration (Base_PCT_) (Eq. 3).

3 $$\:{Base}_{PCT}={RateIn}_{PCT}/{CL}_{PCT}$$

Total body mass and postmenstrual age were included as covariates in the model as maturation of organ function and body size are the most influential for neonatal drug kinetics [18] and therefore were included in the base model *a priori* based on allometric theory and established maturation functions. Allometric scaling using total body mass as a predictor of body size was applied *a priori* to clearance and volume parameters, scaled to a standard value of 70 kg with fixed theory based allometric exponents of 3/4 and 1 respectively [18]. An empirical sigmoidal emax model using postmenstrual age was applied to CL_PCT_ to account for the maturation of elimination processes [19], as shown in Eq. 4.

4 $$\:{F}_{mat,CL}=\:\frac{1}{1+\:{\left(\frac{PMA}{{TM}_{50PCT}}\right)}^{-{Hill}_{PCT}}}$$

where F_mat, CL_ is the variable name used to describe the fraction of adult maturation of clearance, PMA is postmenstrual age expressed in weeks, TM_50PCT_ is the maturation half-time in weeks, and the Hill_PCT_ exponent relates to the slope of the maturation profile.

The NM-TRAN control stream for the imputation scenarios with the final parameter estimates for Scenario 0 is shown in the Supplementary Material.

### Parameter estimation

Data were analysed using non-linear mixed effects models (NONMEM 7.5.1, ICON PLC, Gaithersburg, MA, USA). Population parameter variability was described using exponential models [20]. A proportional error model was used to describe unexplained residual variability of procalcitonin concentrations [20]. The number of parameters and the structural, fixed, and random effects models were kept the same for all scenarios. The initial parameter estimates were the same in all the imputation scenarios. Estimates of the population parameters, covariate effects and variances were obtained using the first order conditional estimation method with interaction, and model equations were integrated using ADVAN = 13 with TOL = 9. The NONMEM objective function value (OFV = minus 2 x log likelihood) was recorded for each scenario replicate. The difference between the OFV from each replicate and the OFV for Scenario 0 was calculated (∆OFV). The average ∆OFV for each scenario was used to select the scenario that best described the observed data. One-way ANOVA with Tukey’s HSD procedure for multiple comparisons was used to test if there were significant differences between the ∆OFVs of the scenarios. Parameter uncertainty for Scenario 0 was assessed using 100 non-parametric bootstrap runs to calculate a relative standard error for each parameter estimate. The average and 95% confidence interval of the ∆OFVs were calculated for each scenario. The OFVs in Scenario 3 were compared pairwise to those in scenarios 1 A, 1B and 2 to investigate the OFV distributions further. Visual predictive checks (VPCs) [21] of the replicate with the median ∆OFV for each imputation scenario were also used to aid scenario selection. The parameter estimates for the run with the median ∆OFV for each scenario were used to simulate the time course of the different scenarios using Berkeley Madonna (Macey & Oster, Berkeley Madonna, Inc) to compare the predicted time course.

## Results

**Fig. 2:**
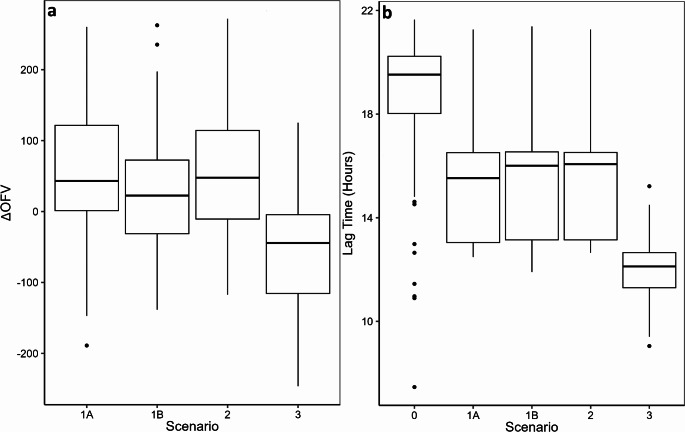
(**a**) Difference in objective function values compared to Scenario 0 (∆OFV). (**b**) Lag time parameter estimates in the scenarios. Scenario 0, without imputation; Scenario 1A, minimum interval of 8 h; Scenario 1B, minimum interval of 12 h; Scenario 2, peak at 1 day; Scenario 3, standard clinical practice at study hospital, 9am start. Median is shown as horizontal line contained within the box, box shows interquartile range, whisker lines indicate range with points indicating outliers. Results for 100 imputed datasets for scenarios 1–3 and 100 non-parametric bootstrap replicates for Scenario 0

**Fig. 3:**
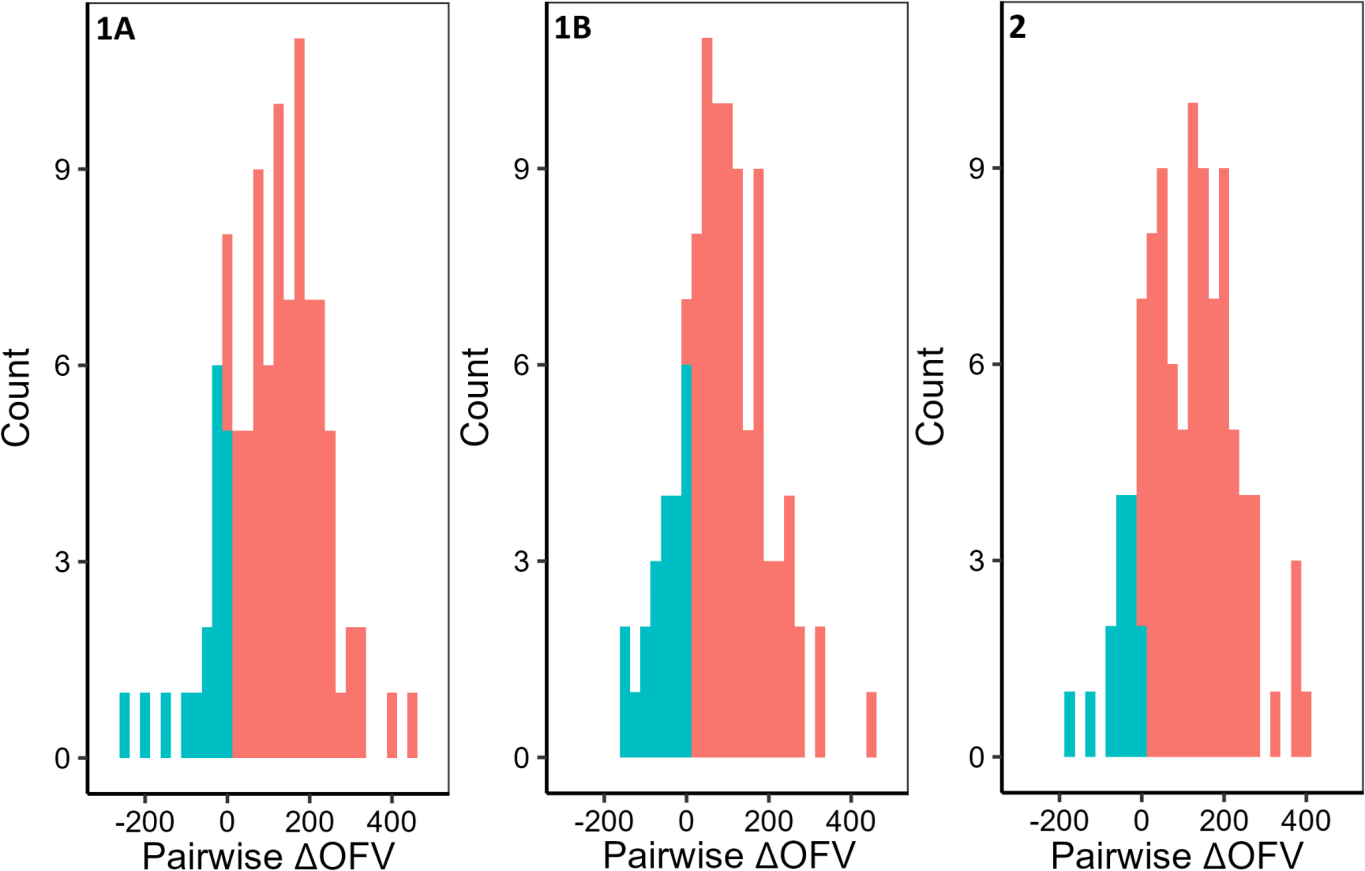
Histograms of pairwise ∆OFV for scenarios 1A, 1B and 2 compared to Scenario 3. Green shows a ∆OFV less than zero, indicating that the investigated scenario is a better fit than Scenario 3, and red shows a ∆OFV greater than zero, indicating a worse fit. Scenario 1A, minimum interval of 8 h; Scenario 1B, minimum interval of 12 h; Scenario 2, peak at 1 day; Scenario 3, standard clinical practice at study hospital, 9am start

The comparison of the scenarios using the average OFV difference compared to Scenario 0 (∆OFV) showed that Scenario 3 (∆OFV: − 62.6) was the best scenario (Table 1). The other imputation scenarios (1A, 1B and 2) had an average ∆OFV greater than zero (Table 1). The ∆OFVs compared to Scenario 0 were significantly lower in Scenario 3 compared to the other scenarios (Fig. 2a; one way ANOVA with Tukey’s HSD; Scenario 1A: *p* < 0.001, Scenario 1B: *p* < 0.05, Scenario 2: *p* < 0.001). The distribution of OFVs compared pairwise to Scenario 3 are skewed to the left for Scenarios 1A and 2, and symmetric for Scenario 1B (Fig. 3). In all cases, Scenario 3 is an improvement (Fig. 3).

**Table 1:** Difference in objective function values compared to scenario 0 (∆OFV) for each imputation scenario. Average, and 95% confidence interval calculated for 100 datasets for each scenario

Scenario	1A	1B	2	3
	As Provided		Peak at 1 day	Clinical Practice
	≥ 8 h	≥ 12 h		
**Average**	51.6	24.3	53.3	-62.6
**95% Confidence Interval**	-131 to 218	-124 to 194	-95.6 to 259	-231 to 60.7

**Table 2:** Parameter estimates for the original dataset (Scenario 0) and each imputation scenario. Average with relative standard error (%) calculated by (bootstrap standard deviation / bootstrap average)*100 for 100 non-parametric bootstraps of Scenario 0 and for 100 imputed datasets for scenarios 1–3

Scenario	0	1A	1B	2	3
	No Imputation	As Provided		Peak at 1 day	Clinical Practice
		≥ 8 h	≥ 12 h		
**RateIn**_**PCT**_ **(µg/h)**	0.00431 (8.97)	0.00471 (3.78)	0.00472 (2.11)	0.00471 (2.79)	0.00466 (3.86)
**CL**_**PCT**_ **(L/h/70kg)**	0.453 (10.1)	0.500 (2.63)	0.502 (2.02)	0.498 (2.49)	0.493 (3.66)
**V**_**PCT**_ **(L/70kg)**	15.0 FIX				
**TM**_**50PCT**_ **(weeks)**	26.5 (12.0)	24.7 (2.71)	24.7 (1.63)	24.6 (2.00)	24.7 (3.04)
**Hill**_**PCT**_	10.3 (18.9)	7.80 (4.05)	7.85 (4.44)	7.87 (2.74)	7.82 (4.88)
**Tel**_**B**_ **(h)**	4.26 (26.7)	5.63 (3.91)	5.64 (4.55)	5.70 (4.84)	5.67 (4.63)
**T**_**LAG**_ **(h)**	18.7 (13.4)	15.3 (14.7)	15.6 (16.4)	15.5 (15.0)	12.0 (9.60)
**SLOPE**_**B**_	77.6 (20.8)	77.3 (6.77)	77.4 (7.81)	77.9 (5.03)	75.4 (6.68)
**Proportional RUV**	0.343 (12.2)	0.295 (3.26)	0.295 (2.11)	0.296 (3.38)	0.294 (4.37)

RateIn_PCT_: Procalcitonin production rate, CL_PCT_: Procalcitonin clearance, V_PCT_: Procalcitonin volume of distribution, TM_50PCT_: Procalcitonin maturation half time in postmenstrual weeks, Hill_PCT_: Hill exponent for sigmoidal maturation function, Tel_B_: Elimination half life of birth effect, T_LAG_: Lag time between of birth event and the procalcitonin concentration increase, SLOPE_B_: proportionality constant between procalcitonin production rate and birth event concentration, Proportional RUV: Proportional error estimate for procalcitonin concentration

Table 2 shows that the Scenario 0 bootstrap had a 1.5 fold distribution of maturation half times (TM_50PCT_; 95% CI: 19.2–30.1 weeks) including postmenstrual ages outside those seen in the dataset (19.2 weeks). The bootstrap average Hill_PCT_ parameter estimate was 10.3 in Scenario 0 with a ~ 2 fold distribution of values (95% CI: 4.99–11.7) (Table 2). The average half-life of the birth effect stimulus (Tel_B_) in Scenario 0 (4.26 h) was lower than in the other imputation scenarios (averages: 5.63–5.70 h; Table 2). The average estimated lag time varied 1.6 fold from 12.0 h in Scenario 3 to 18.7 h in Scenario 0 (Table 2). Further details of the distributions of parameter estimates for each scenario are described further in the Supplementary Material.

**Fig. 4:**
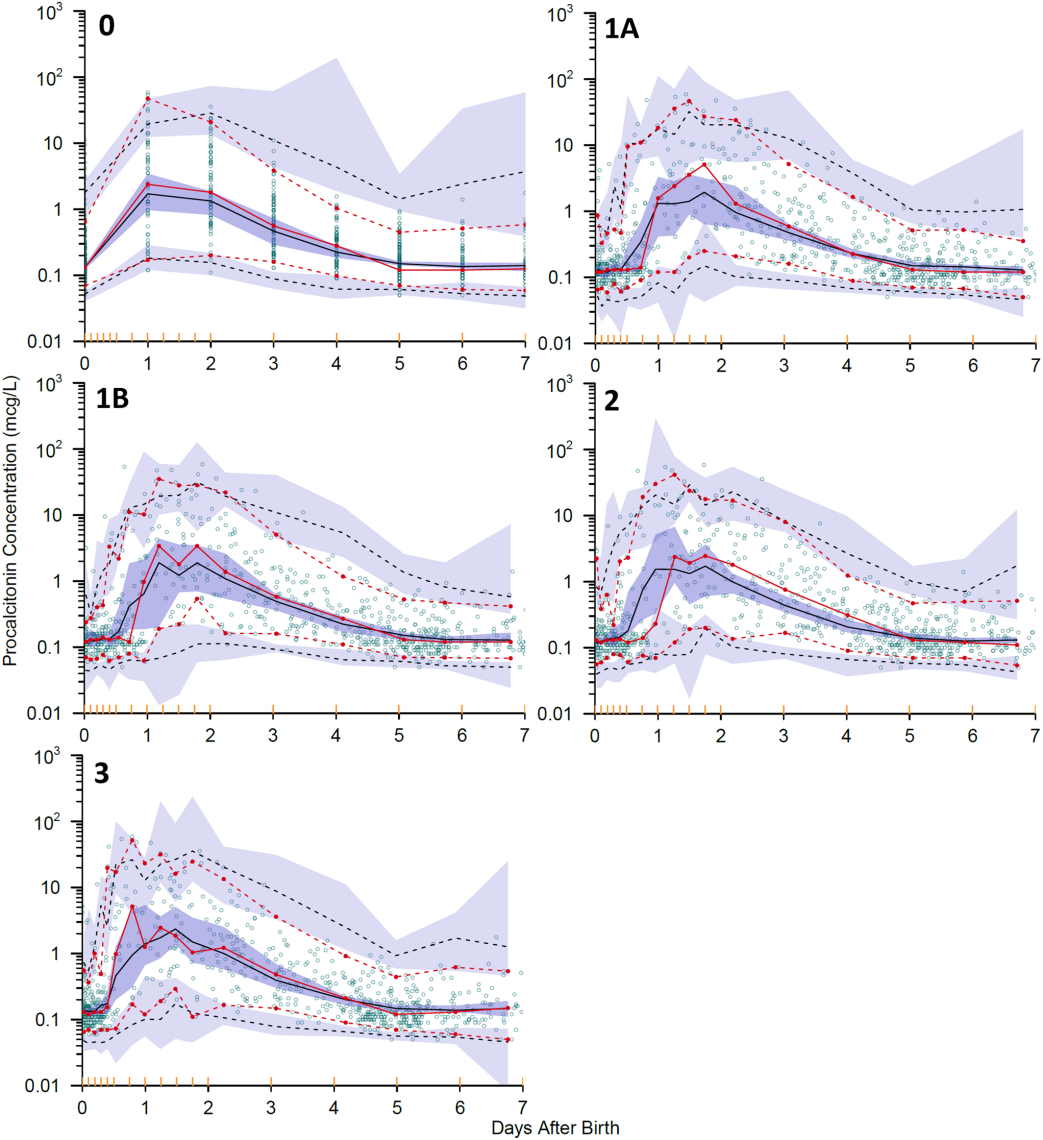
Visual predictive check (VPC) of model predicted and observed procalcitonin concentration (mcg/L) in the first seven postnatal days. Scenario 0, without imputation; Scenario 1A, minimum interval of 8 h; Scenario 1B, minimum interval of 12 h; Scenario 2, peak at 1 day; Scenario 3, clinical practice at study hospital, 9am start. For Scenario 0 the VPC is from the run used for the bootstraps. For the imputation scenarios VPCs are shown for a run associated with a ∆OFV closest to the median ∆OFV for that scenario. Red lines are observed data and black lines are predicted by the simulation. Solid lines are medians of the observed and predicted values, dashed lines are 5th and 95th percentiles. Shaded areas are 95% confidence intervals for each of the prediction percentiles obtained by simulation

Representative VPCs for imputation replicates with a ∆OFV closest to the median ∆OFV for each scenario are shown in Fig. 4. The predicted procalcitonin concentrations for Scenarios 1B and 3 were closer to the observed concentrations than those for Scenarios 1A and 2 (Fig. 4, panel 1A − 3). The VPC for Scenario 3 shows a median prediction that does not show a systematic difference (3 or more consecutive prediction-observation pairs) unlike all other scenarios. Based on the VPC this is the scenario with the best fit to the time course of procalcitonin observations.

**Fig. 5:**
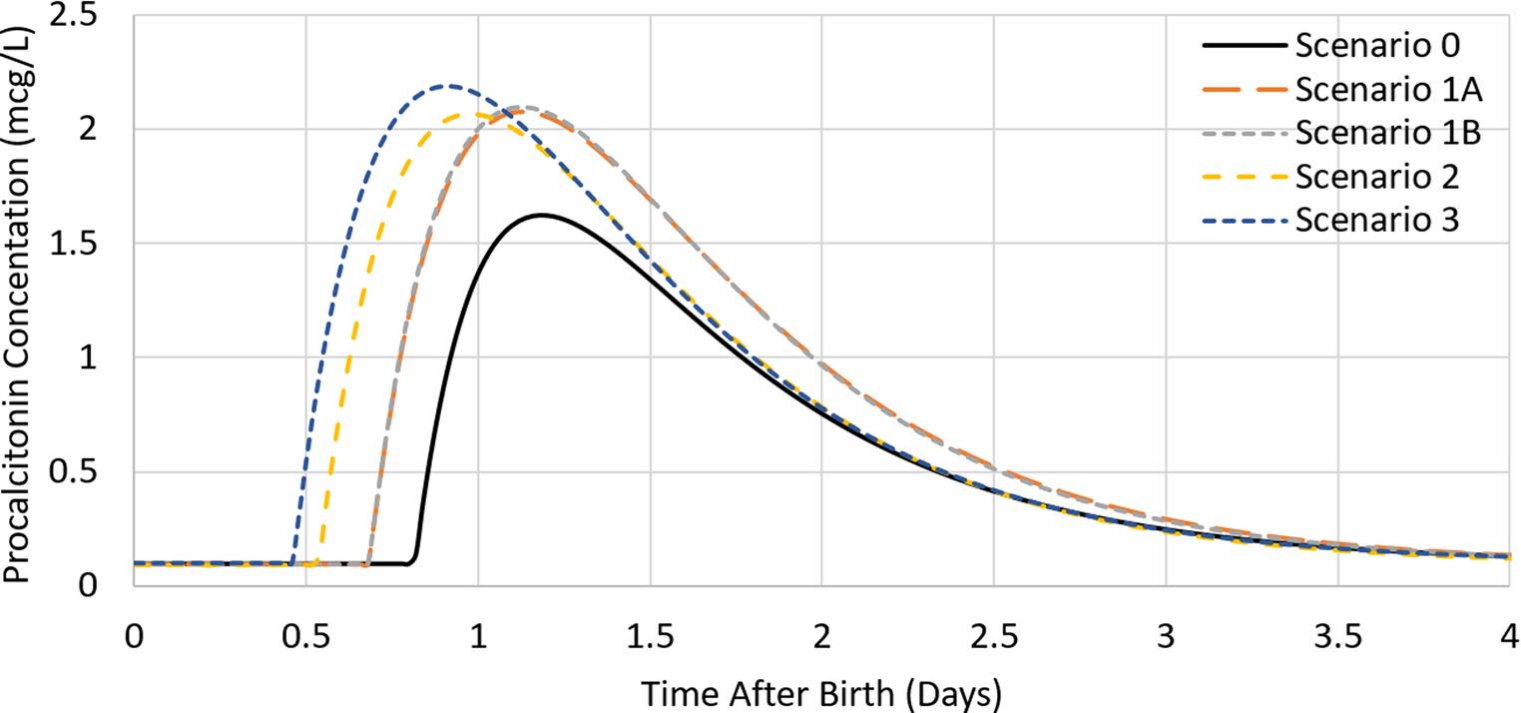
Simulated time course of procalcitonin concentrations (mcg/L) following birth for four postnatal days using the parameter estimates from a run with a median ∆OFV for each scenario. Scenario 0: without imputation; Scenario 1A: minimum interval of 8 h; Scenario 1B: minimum interval of 12 h; Scenario 2: peak at 1 day; Scenario 3: standard clinical practice at study hospital, 9am start. Male neonate of 40 weeks postmenstrual age and 3.36 kg at birth based on estimated weight equation in [22]

Parameter estimates from a run with a ∆OFV closest to the median ∆OFV were used to simulate the time course of procalcitonin concentrations following birth. Procalcitonin concentrations after birth peaked earliest for Scenario 3 (22.1 h) and latest for Scenario 0 (28.6 h) (Fig. 5; Table 3). The peak procalcitonin concentration was much lower in Scenario 0, compared to the other scenarios (Fig. 5; Table 3).

**Table 3:** Procalcitonin peak time and concentration following birth for each scenario, as shown in Fig. 5 using the parameter estimates from a run with the median ∆OFV. Male neonate of 40 weeks postmenstrual age and 3.36 kg at birth based on estimated weight equation in [22]

Scenario	0	1 A	1B	2	3
	No Imputation	As Provided		Peak at 1 day	Clinical Practice
		≥ 8 h	≥ 12 h		
Time of Peak (h)	28.6	26.9	26.9	23.5	22.1
Peak Concentration (mcg/L)	1.62	2.09	2.09	2.07	2.19

Scenario 3 was further explored with a first measurement interval between 8:00 and 10:00, rather than strictly at 9:00. This interval more closely aligns with expected clinical practice. With this variation, parameter estimates were within 5% of those in the original Scenario 3 and the average lag time decreased slightly from 12.0 h to 11.5 h. This indicates that this Scenario 3 variation did not produce a noticeable improvement.

## Discussion

It is common for studies on the concentration of procalcitonin to report data by day without including the time of sampling [4, 7, 23]. In the dataset used for our study, within-day clock times of birth and procalcitonin sampling were not recorded and could not be obtained retrospectively, so we used an imputation approach to improve the resolution of the sampling time. A model based approach was used to investigate imputation scenarios on the description of the time course of procalcitonin.

Multiple imputation is commonly used when covariate data is missing [24]. Imputation is repeated multiple times and each dataset is analysed separately. It is superior to a single imputation as uncertainty can be included so that the imputed value is not considered the true value [24]. In this study random error was introduced in the imputation replicates assuming a uniform distribution of birth and sample times. Imputation methods typically impute missing covariates, while we imputed a missing part of the independent variable time after birth.

In our study, imputation spreads the observation times across the day, which is a more accurate description of clinical sample collection times than reporting observations by study day. Overall, compared with not using imputation (Scenario 0), Scenario 3 had the best performance when evaluated by both ∆OFV and VPC.

The peak procalcitonin concentration was much lower in Scenario 0, which has no samples between 24 and 48 h after birth. This likely reflects a misinterpretation of the observed concentrations because the lack of samples in this period means information is missing on the time and magnitude of peak concentration. Also, all concentrations from the first postnatal day are grouped into the 24 h time point, which will influence the parameter estimates leading to a later and lower peak concentration (Fig. 5).

Scenario 3 showed an improvement when the distribution of OFVs were compared pairwise with the other scenarios (Fig. 3) supporting the box-whisker plot statistics in Fig. 2. Scenario 3 had the earliest peak, around 22 h after birth, similar to other reports (21–24 h) with timed observations during the day [15, 25]. This shows that knowledge of typical clinical practice is a good way to inform imputation scenarios. The scenarios were imputed based on clinical sampling procedures. If a biomarker is known to have diurnal variation then this could be considered as part of an imputation scenario but, to our knowledge, diurnal variation has not been described for procalcitonin.

Some maturation half times (TM_50PCT_) from the bootstrap of Scenario 0 were lower than postmenstrual ages in the dataset, and the average Hill exponent was large (10.3), implying an abrupt square wave change in maturation from 0 to adult values which does not seem biologically plausible. This highlights that without imputation, it is difficult to accept the parameter estimates associated with the time course. Based on Scenario 0 with a short average half life of the birth effect (Tel_B_) 4.26 h compared with Scenario 3 (5.67 h) and long average lag time (18.7 h) compared with Scenario 3 (12.0 h) (Table 2) reveals the loss of information based on just using study day rather than clock time.

The random effects in Scenario 0 come from re-sampling based on observations (procalcitonin) while in the other scenarios the random effects come from resampling from the independent variable (clock time). The sources of random effects are different, so no attempt is made to apply tests such as the likelihood ratio test. The OFV distributions shown in Fig. 2a are provided to give a graphical indication of the goodness of fit of Scenarios 1 to 3 compared with Scenario 0. The purpose is to illustrate that the scenarios with imputed clock times and a lag time in the model appear to give a better description of the procalcitonin concentrations.

Within each scenario the 95% confidence interval for the lag time estimate did not include zero (Fig. 2b), showing that the lag time is greater than zero. The presence of a lag time between the time of the birth event and a detectable change in C_PCT_ was expected. Clinical studies have shown that it takes 3–6 h for procalcitonin concentrations to increase after bacterial stimuli (measured at 3, 6 and 24 h) [26], endotoxin administration (measured at 2 h intervals for 8 h) [14] or surgical trauma (measured at 6 h post-surgery) [27]. Other studies have reported that procalcitonin concentrations are low at birth and take time to rise, but the delay was not quantified [15, 28]. Scenario 3 has the shortest average lag time (12.0 h) compared to the other imputation scenarios (averages: 15.3–18.7 h; Table 2), however, this is still much longer than the delay seen following endotoxin administration or infection (3–4 h) [11, 29]. The lag time parameter estimate is design dependent and appropriate sampling times are needed to inform the onset of the rise. The use of imputed clocktimes explains why the imputation scenarios 1 to 3 have shorter lag times than Scenario 0 (no imputation). It should be noted that the lag time does not directly reflect a physiological process but is an approximation of a delay that might be better described by a transit chain process [30].

It is reassuring that Scenario 3 was detected as the most appropriate imputation scenario because it aligns most closely with what occurs in clinical practice. In an attempt to further align with clinical practice, Scenario 3 was extended so that the first measurement time each day was between 8:00 and 10:00, however this did not significantly change parameter estimates such as lag time. This study demonstrates that the use of clock time, informed by clinical practice, and study day improves imputation over other approaches when the exact sampling time is unavailable. As Scenario 3 was based on clinical practice at the study hospital, and not on properties of procalcitonin or a sampling procedure unique to this biomarker, this scenario could be used for other biomarkers collected within the same blood sample.

In the original study, the samples were collected as part of routine patient care and the data was collected for a purpose other than pharmacometric modelling, so sampling times were likely unimportant for the original purpose. However, when biomarker concentrations are expected to change rapidly, the direction of change and the duration over which it occurs is likely important, so recording sample clock times and the study day is a good policy for clinical investigators working in this area.

A limitation of this approach is that the results of the imputation scenarios are based on the assumptions made during the imputation process. Therefore, assumptions have been centred around what is clinically feasible for sample collection. A potential limitation is that the imputation approach may not completely address the lack of clock times because actual clock times are unknown.

An alternative approach we could have taken is to complete an entirely simulation based imputation study in which the clock times were determined, then removed in order to investigate the appropriateness of the different imputation scenarios. However, any results for this simulation would still be speculative as the dataset true clock times are not known.

We have applied the imputation approach to studies of procalcitonin [31, 32] and of c-reactive protein following birth and/or surgery where clock times were missing [31]. In all cases, the imputed datasets based on sampling times informed by clinical practice have been shown to improve the interpretation of the time course of biomarker concentrations following these events. Our analysis presents a framework for imputing clock times that could be used in other similar studies where times are missing.

## Electronic supplementary material

Below is the link to the electronic supplementary material.

Supplementary Material 1

## Data Availability

Data was obtained from Fukuzumi et al. [16] and any requests for original data should be addressed to the authors.

## References

[1] McDonough MH, Stocker SL, Kippin TE, Meiring W, Plaxco KW (2023) Using seconds-resolved pharmacokinetic datasets to assess pharmacokinetic models encompassing time-varying physiology. Br J Clin Pharmacol. 10.1111/bcp.15756 37186478 10.1111/bcp.15756 PMC10799768

[2] Hope WW, Petraitis V, Walsh TJ (2008) Experimental Design considerations in Pharmacokinetic studies. Preclinical Development Handbook: ADME and Biopharmaceutical Properties. John Wiley & Sons, Inc, Hoboken, NJ, USA, pp 1059–1068. 10.1002/9780470249031

[3] Marchini G, Berggren V, Djilali-Merzoug R, Hansson LO (2000) The birth process initiates an acute phase reaction in the fetus-newborn infant. Acta Paediatr 89(9):1082–1086. 10.1080/713794557 11071089 10.1080/713794557

[4] Lee J, Bang YH, Lee EH, Choi BM, Hong YS (2017) The influencing factors on procalcitonin values in newborns with noninfectious conditions during the first week of life. Korean J Pediatr 60(1):10–16. 10.3345/kjp.2017.60.1.10 28203255 10.3345/kjp.2017.60.1.10 PMC5309319

[5] Csontos C, Foldi V, Palinkas L, Bogar L, Roth E, Weber G, Lantos J (2010) Time course of pro- and anti-inflammatory cytokine levels in patients with burns–prognostic value of interleukin-10. Burns 36(4):483–494. 10.1016/j.burns.2009.10.009 20045261 10.1016/j.burns.2009.10.009

[6] Hur J, Yang HT, Chun W, Kim JH, Shin SH, Kang HJ, Kim HS (2015) Inflammatory cytokines and their prognostic ability in cases of Major burn Injury. Ann Lab Med 35(1):105–110. 10.3343/alm.2015.35.1.105 25553289 10.3343/alm.2015.35.1.105 PMC4272939

[7] D’Souza S, Guhadasan R, Jennings R, Siner S, Paulus S, Thorburn K, Chesters C, Downey C, Baines P, Lane S, Carrol E (2019) Procalcitonin and other common biomarkers do not reliably identify patients at risk for bacterial infection after congenital heart surgery. Pediatr Crit Care Med 20(3):243–251. 10.1097/PCC.0000000000001826 30575697 10.1097/PCC.0000000000001826

[8] Zhang WR, Garg AX, Coca SG, Devereaux PJ, Eikelboom J, Kavsak P, McArthur E, Thiessen-Philbrook H, Shortt C, Shlipak M, Whitlock R, Parikh CR, Consortium T-A (2015) Plasma IL-6 and IL-10 concentrations predict AKI and Long-Term mortality in adults after cardiac surgery. J Am Soc Nephrol 26(12):3123–3132. 10.1681/ASN.2014080764 25855775 10.1681/ASN.2014080764 PMC4657830

[9] Maier B, Lefering R, Lehnert M, Laurer HL, Steudel WI, Neugebauer EA, Marzi I (2007) Early versus late onset of multiple organ failure is associated with differing patterns of plasma cytokine biomarker expression and outcome after severe trauma. Shock 28(6):668–674. 10.1097/shk.0b013e318123e64e 18092384

[10] Fullerton JN, Segre E, De Maeyer RP, Maini AA, Gilroy DW (2016) Intravenous Endotoxin Challenge in Healthy Humans: An Experimental Platform to Investigate and Modulate Systemic Inflammation. J Vis Exp (111). 10.3791/53913 10.3791/53913 PMC4942172 27213711

[11] Brunkhorst FM, Heinz U, Forycki ZF (1998) Kinetics of procalcitonin in iatrogenic sepsis. Intensive Care Med 24(8):888–889. 10.1007/s001340050683 9757936 10.1007/s001340050683

[12] Bohnhorst B, Lange M, Bartels DB, Bejo L, Hoy L, Peter C (2012) Procalcitonin and valuable clinical symptoms in the early detection of neonatal late-onset bacterial infection. Acta Paediatr 101(1):19–25. 10.1111/j.1651-2227.2011.02438.x 21824193 10.1111/j.1651-2227.2011.02438.x

[13] Auriti C, Fiscarelli E, Ronchetti MP, Argentieri M, Marrocco G, Quondamcarlo A, Seganti G, Bagnoli F, Buonocore G, Serra G, Bacolla G, Mastropasqua S, Mari A, Corchia C, Prencipe G, Piersigilli F, Rava L, Di Ciommo V (2012) Procalcitonin in detecting neonatal nosocomial sepsis. Arch Dis Child Fetal Neonatal Ed 97(5):F368–370. 10.1136/fetalneonatal-2010-194100 22933097 10.1136/fetalneonatal-2010-194100

[14] Dandona P, Nix D, Wilson MF, Aljada A, Love J, Assicot M, Bohuon C (1994) Procalcitonin increase after endotoxin injection in normal subjects. J Clin Endocrinol Metab 79(6):1605–1608. 10.1210/jcem.79.6.7989463 7989463 10.1210/jcem.79.6.7989463

[15] Chiesa C, Natale F, Pascone R, Osborn JF, Pacifico L, Bonci E, De Curtis M (2011) C reactive protein and procalcitonin: reference intervals for preterm and term newborns during the early neonatal period. Clin Chim Acta 412(11–12):1053–1059. 10.1016/j.cca.2011.02.020 21338596 10.1016/j.cca.2011.02.020

[16] Fukuzumi N, Osawa K, Sato I, Iwatani S, Ishino R, Hayashi N, Iijima K, Saegusa J, Morioka I (2016) Age-specific percentile-based reference curve of serum procalcitonin concentrations in Japanese preterm infants. Sci Rep 6(1):1–6 27033746 10.1038/srep23871 PMC4817150

[17] Emerge Health New Zealand Ltd (2018) Miacalcic ampoules. New Zealand data sheet. Medsafe, Auckland, New Zealand

[18] Anderson BJ, Holford NH (2008) Mechanism-based concepts of size and maturity in pharmacokinetics. Annu Rev Pharmacol Toxicol 48:303–332. 10.1146/annurev.pharmtox.48.113006.094708 17914927 10.1146/annurev.pharmtox.48.113006.094708

[19] Holford N, Heo YA, Anderson B (2013) A Pharmacokinetic Standard for Babies and adults. J Pharm Sci 102(9):2941–2952. 10.1002/jps.23574 23650116 10.1002/jps.23574

[20] Owen JS (2014) Introduction to population pharmacokinetic/pharmacodynamic analysis with nonlinear mixed effects models. Hoboken, N.J.: Wiley. 2014 10.1038/psp.2014.51 PMC5684957 25531560

[21] Nguyen TH, Mouksassi MS, Holford N, Al-Huniti N, Freedman I, Hooker AC, John J, Karlsson MO, Mould DR, Perez Ruixo JJ, Plan EL, Savic R, van Hasselt JG, Weber B, Zhou C, Comets E, Mentre F, Model Evaluation Group of the International Society of Pharmacometrics Best Practice C (2017) Model evaluation of continuous data pharmacometric models: Metrics and Graphics. CPT Pharmacometrics Syst Pharmacol 6(2):87–109. 10.1002/psp4.12161 27884052 10.1002/psp4.12161 PMC5321813

[22] Sumpter AL, Holford NH (2011) Predicting weight using postmenstrual age–neonates to adults. Paediatr Anaesth 21(3):309–315. 10.1111/j.1460-9592.2011.03534.x 21320235 10.1111/j.1460-9592.2011.03534.x

[23] Sponholz C, Sakr Y, Reinhart K, Brunkhorst F (2006) Diagnostic value and prognostic implications of serum procalcitonin after cardiac surgery: a systematic review of the literature. Crit Care 10(5):R145. 10.1186/cc5067 17038199 10.1186/cc5067 PMC1751067

[24] Johansson AM, Karlsson MO (2013) Comparison of methods for handling missing covariate data. AAPS J 15(4):1232–1241. 10.1208/s12248-013-9526-y 24022319 10.1208/s12248-013-9526-y PMC3787222

[25] Chiesa C, Panero A, Rossi N, Stegagno M, Giusti MD, Osborn JF, Pacifico L (1998) Reliability of procalcitonin concentrations for the diagnosis of sepsis in critically ill neonates. Clin Infect Dis 26(3):664–672 9524841 10.1086/514576

[26] Petitjean S, Assicot M, Bohuon C (1994) Etude De l’immunoréactivité calcitonin-like Au cours des processus infectieux. Immunol Biol Spec 9(5):302–307

[27] Beghetti M, Rimensberger PC, Kalangos A, Habre W, Gervaix A (2003) Kinetics of procalcitonin, interleukin 6 and C-reactive protein after cardiopulmonary-bypass in children. Cardiol Young 13(2):161–167. 10.1017/s1047951103000301 12887072 10.1017/s1047951103000301

[28] Assumma M, Signore F, Pacifico L, Rossi N, Osborn JF, Chiesa C (2000) Serum procalcitonin concentrations in term delivering mothers and their healthy offspring: a longitudinal study. Clin Chem 46(10):1583–1587 11017935

[29] Meisner M (1999) Procalcitonin: Erfahrungen Mit Einer Neuen Meßgröße für bakterielle infektionen und systemische inflammation. Laboratoriumsmedizin 23(5):263–272. 10.1515/labm.1999.23.5.263

[30] Friberg LE, Henningsson A, Maas H, Nguyen L, Karlsson MO (2002) Model of Chemotherapy-Induced Myelosuppression with parameter consistency across drugs. J Clin Oncol 20:4713–4721 12488418 10.1200/JCO.2002.02.140

[31] Bokor A, Holford N, Hannam J (2023) Time course of biomarkers of inflammation following birth and surgery. Paper presented at the Australasian Society of Clinical and Experimental Pharmacologists and Toxicologists, Sydney, Australia

[32] Bokor A, Holford N, Hannam J (2023) Time Course of Procalcitonin Concentration Following Birth and Surgery. Paper presented at the Population Approach Group of Australia & New Zealand, Adelaide, Australia

