# Supplementary material for: Imputation of missing clock times – application to procalcitonin concentration time course after birth
Source: J Pharmacokinet Pharmacodyn. 2025 Mar 18;52(2):20. doi: 10.1007/s10928-025-09965-8 (PMC11920349; doi:10.1007/s10928-025-09965-8)
Supplement: Supplementary file 1 — Supplementary Material 1 [file 10928_2025_9965_MOESM1_ESM.docx]

**Supplementary Data**

**Imputation of Missing Clock Times – Application to Procalcitonin Concentration Time Course after Birth. JPKPD, Abigail J Bokor, Nick Holford, Jacqueline A Hannam; Corresponding Author: Jacqueline A Hannam ; Department of Pharmacology & Clinical Pharmacology, The University of Auckland, Auckland, New Zealand; j.hannam@auckland.ac.nz**

**Supplementary Text 1** NM-TRAN control stream with final estimates replacing initial estimates.

$PROB CHANGES IN PCT AFTER BIRTH, HEALTHY NEONATES

$INPUT

ID ; Subject identifier

TIME ; Time since birth (days), Scenario 0: integer days, Scenario 1 -3: decimal days as imputed

PMAWB ; Postmenstrual age at birth (weeks)

PMAW ; Postmenstrual age (weeks)

PNAD ; Postnatal age (days), Scenario 0: integer days, Scenario 1 -3: decimal days

WTB ; Weight at birth (g)

CMT ; NONMEM Compartment number

EVID ; NONMEM Event identifier

MDV ; NONMEM Missing dependent variable

DVID ; Dependent variable identifier

DV ; NONMEM Dependent variable value

AMT ; Hypothetical amount at birth

EWT ; Estimated weight (g (based on Sumpter & Holford, 2011)

CWT ; Current weight (g)

EWTRATIO ; Ratio at last observed weight between estimated weight and observed weight

$DATA ..\Datafiles\20220709_NoImp_Mor.CSV IGNORE #

$ESTIM MAXEVAL=9999 NSIG=3 SIGL=9 METHOD=CONDITIONAL INTERACTION PRINT=1 NOABORT

$COV

;; ----------- PCT compartment parameters ------------- ;;

$THETA

(0,0.103,) ; POP_RATEIN , mcg/day

(0,10.5, 500) ; POP_CL, L/day/70kg

15. FIX ; POP_VC, L/70kg

;; ----------- Birth compartment parameters ------------- ;;

(0,0.149,5 ) ; POP_TELB, days

(0,83.,300) ; POP_SLOPEB

(0,0.826,5) ; POP_TLAGB, days

;; ----------- Age effect parameter ------------- ;;

(0,25.2,200) ; POP_TM50 , weeks

(0.01,11.7,20) ; Hill

;; ----------- BSV parameters ------------- ;;

$OMEGA BLOCK(5)

0.276 ; PPV_RATEIN

0.125 0.0699 ; PPV_CL

0.144 0.0416 2.37 ; PPV_SLOPEB

0.0102 0.00467 0.0609 0.00439 ; PPV_TLAGB

0.0156 0.019 -0.315 -0.00915 0.112 ; PPV_TM50

$OMEGA

0 FIX ; PPV_TELB

;; ----------- RUV parameters ------------- ;;

$THETA 0 FIX ; RUV_SD ;mcg/L

$THETA (0,0.314, ) ; RUV_CV

$SIGMA 1. FIX ; EPS1

$SUBR ADVAN13 TOL=9

$MODEL

COMP (BIRTH) ; Birth effect compartment

COMP (PCT) ; PCT compartment

$PK

IF (NEWIND.EQ.0) THEN ; First record of dataset, initialize constants

LN2=LOG(2)

PWRCL=3/4

ENDIF

;; ----- Effect of gestational age on baseline PCT ------ ;;

TM50=THETA(7)*EXP(ETA(5))

FMAT=1/(1+((PMAW/TM50)**(-THETA(8))))

WTDF=0 ; To calculate WT based on birth weight below

IF (CWT.LE.0.AND.WTB.LE.0) THEN

WTG=EWT*(1+EWTRATIO)

ELSEIF (CWT.LE.0) THEN

WTDF=(WTB-EWTB)/EWTB

WTG=EWT*(1+WTDF)

ELSE

WTG=CWT

ENDIF

IF (TIME.LE.5.AND.STUDY.EQ.1) THEN ; use birth weight

WT=WTB/1000; g -> kg

ELSE

WT=WTG/1000 ; g -> kg

ENDIF

FSZ=WT/70

FSZCL=FSZ**PWRCL

;; ---- Birth compartment & stimulatory effect on PCT synthesis ---- ;;

TELB=THETA(4)*EXP(ETA(6))

KELB=LN2/TELB

SLOPEBE=THETA(5)*EXP(ETA(3))

VB=1

TLAGB=THETA(6)*EXP(ETA(4))

ALAG1 = TLAGB

;; --------- PCT parameters ----- ;;

VC=THETA(3)*FSZ

CL=THETA(2)*FSZCL*EXP(ETA(2))*FMAT

RATEIN_PCT=THETA(1)*EXP(ETA(1))

BASE = RATEIN_PCT/CL ; mg/L = mg/day / L/day

;; ----------------- Initialise compartments ------------- ;;

BASE_AMT_PCT=BASE*VC ; mg = mg/L x L

IF (A_0FLG.EQ.1) THEN

A_0(1) = 0 ; Initial amount in birth effect compartment

A_0(2)=BASE_AMT_PCT ; Initial amount in PCT compartment

ENDIF

$DES

CONCBE=A(1)/VB ; birth effect compartment

DCP=A(2)/VC ; PCT compartment

;; Birth Effect Compartment

IF (CONCBE.LE.0.OR.T.LT.TLAGB)THEN

SYNB=1

ELSE

SYNB=1 + (SLOPEBE * CONCBE)

ENDIF

;; Differential Equations

DADT(1) = -KELB*A(1) ;; Birth effect compartment

DADT(2) = (RATEIN_PCT*SYNB)-(DCP*CL);; PCT compartment

$ERROR

PCT=A(2)/VC

PROP=PCT*THETA(10)

ADD=THETA(9) SD=SQRT((PROP*PROP) + (ADD*ADD))

IF (DVID.EQ.2) THEN

FLAG=0

W=SD

Y=PCT + W*ERR(1)

ENDIF

$TABLE ID TIME DV Y PCT WTB WT MDV DVID CONCBE SYNB

NOPRINT ONEHEADER FILE=turnover_pct_base_final.fit

**Supplementary Table 1** Parameter estimate table for Scenario 0, no imputation, for 100 non-parametric bootstraps. RSE: relative standard error calculated by (bootstrap standard deviation / bootstrap average)*100.

| **Name** | **Description** | **Units** | **Original Estimate** | **Average** | **95% Confidence Interval** | **RSE (%)** |
| --- | --- | --- | --- | --- | --- | --- |
| RateIn_PCT_ | PCT production rate | µg/h | 0.00429 | 0.00431 | 0.00359 – 0.00513 | 8.97 |
| CL_PCT_ | PCT clearance | L/h/70kg | 0.438 | 0.453 | 0.394 – 0.519 | 10.1 |
| V_PCT_ | PCT volume of distribution | L/70kg | 15 FIX | 15 FIX | | |
| TM_50PCT_ | Maturation half life | weeks | 25.2 | 26.5 | 19.2 – 30.1 | 12.0 |
| Hill_PCT_ | Hill exponent | - | 11.7 | 10.3 | 4.99 – 11.7 | 18.9 |
| Tel_B_ | Elimination half life of birth event | h | 3.58 | 4.26 | 3.05 – 7.07 | 26.7 |
| T_LAG_ | Lag time | h | 19.8 | 18.7 | 11.2 – 21.3 | 13.4 |
| SLOPE_B_ | Linear relationship between birth event concentration and PCT production rate | - | 83.0 | 77.6 | 39.4 – 105 | 20.8 |
| Proportional RUV | Proportional error | - | 0.314 | 0.343 | 0.281 – 0.440 | 12.2 |

**Supplementary Table 2** Parameter estimate table for Scenario 1A, minimum intervals between procalcitonin observations of 8 h, for 100 imputed datasets. RSE: relative standard error calculated by (bootstrap standard deviation / bootstrap average)*100.

| **Name** | **Description** | **Units** | **Scenario 0 Estimate** | **Average** | **95% Confidence Interval** | **RSE (%)** |
| --- | --- | --- | --- | --- | --- | --- |
| RateIn_PCT_ | PCT production rate | µg/h | 0.00429 | 0.00471 | 0.00439 – 0.00511 | 3.78 |
| CL_PCT_ | PCT clearance | L/h/70kg | 0.438 | 0.500 | 0.475 – 0.534 | 2.63 |
| V_PCT_ | PCT volume of distribution | L/70kg | 15 FIX | 15 FIX | | |
| TM_50PCT_ | Maturation half life | weeks | 25.2 | 24.7 | 23.8 – 26.3 | 2.71 |
| Hill_PCT_ | Hill exponent | - | 11.7 | 7.80 | 6.93 – 8.47 | 4.05 |
| Tel_B_ | Elimination half life of birth event | h | 3.58 | 5.63 | 5.19 – 6.22 | 3.91 |
| T_LAG_ | Lag time | h | 19.8 | 15.3 | 12.7 – 21.2 | 14.7 |
| SLOPE_B_ | Linear relationship between birth event concentration and PCT production rate | - | 83.0 | 77.3 | 61.4 – 83.1 | 6.77 |
| Proportional RUV | Proportional error | - | 0.314 | 0.295 | 0.281 – 0.316 | 3.26 |

**Supplementary Table 3** Parameter estimate table for Scenario 1B, minimum intervals between procalcitonin observations of 12 h, for 100 imputed datasets. RSE: relative standard error calculated by (bootstrap standard deviation / bootstrap average)*100.

| **Name** | **Description** | **Units** | **Scenario 0 Estimate** | **Average** | **95% Confidence Interval** | **RSE (%)** |
| --- | --- | --- | --- | --- | --- | --- |
| RateIn_PCT_ | PCT production rate | µg/h | 0.00429 | 0.00472 | 0.00460 – 0.00494 | 2.11 |
| CL_PCT_ | PCT clearance | L/h/70kg | 0.438 | 0.502 | 0.485 – 0.531 | 2.02 |
| V_PCT_ | PCT volume of distribution | L/70kg | 15 FIX | 15 FIX | | |
| TM_50PCT_ | Maturation half life | weeks | 25.2 | 24.7 | 24.1 – 25.9 | 1.63 |
| Hill_PCT_ | Hill exponent | - | 11.7 | 7.85 | 6.98 – 8.63 | 4.44 |
| Tel_B_ | Elimination half life of birth event | h | 3.58 | 5.64 | 5.09 – 6.10 | 4.55 |
| T_LAG_ | Lag time | h | 19.8 | 15.6 | 12.6 – 21.2 | 16.4 |
| SLOPE_B_ | Linear relationship between birth event concentration and PCT production rate | - | 83.0 | 77.4 | 63.7 – 82.3 | 7.81 |
| Proportional RUV | Proportional error | - | 0.314 | 0.295 | 0.279 – 0.308 | 2.11 |

**Supplementary Table 4** Parameter estimate table for Scenario 2, procalcitonin observations peak on postnatal day 1, for 100 imputed datasets. RSE: relative standard error calculated by (bootstrap standard deviation / bootstrap average)*100.

| **Name** | **Description** | **Units** | **Scenario 0 Estimate** | **Average** | **95% Confidence Interval** | **RSE (%)** |
| --- | --- | --- | --- | --- | --- | --- |
| RateIn_PCT_ | PCT production rate | µg/h | 0.00429 | 0.00471 | 0.00448 – 0.00502 | 2.79 |
| CL_PCT_ | PCT clearance | L/h/70kg | 0.438 | 0.498 | 0.471 – 0.517 | 2.49 |
| V_PCT_ | PCT volume of distribution | L/70kg | 15 FIX | 15 FIX | | |
| TM_50PCT_ | Maturation half life | weeks | 25.2 | 24.6 | 23.3 – 25.2 | 2.00 |
| Hill_PCT_ | Hill exponent | - | 11.7 | 7.87 | 7.50 – 8.42 | 2.74 |
| Tel_B_ | Elimination half life of birth event | h | 3.58 | 5.70 | 5.44 – 6.36 | 4.84 |
| T_LAG_ | Lag time | h | 19.8 | 15.5 | 12.7 – 21.2 | 15.0 |
| SLOPE_B_ | Linear relationship between birth event concentration and PCT production rate | - | 83.0 | 75.4 | 63.0 – 81.8 | 5.03 |
| Proportional RUV | Proportional error | - | 0.314 | 0.296 | 0.280 – 0.319 | 3.38 |

**Supplementary Table 5** Parameter estimate table for Scenario 3, standard clinical practice at the study hospital, first observation of each day at 9:00, for 100 imputed datasets. RSE: relative standard error calculated by (bootstrap standard deviation / bootstrap average)*100.

| **Name** | **Description** | **Units** | **Scenario 0** | **Average** | **95% Confidence Interval** | **RSE (%)** |
| --- | --- | --- | --- | --- | --- | --- |
| RateIn_PCT_ | PCT production rate | µg/h | 0.00429 | 0.00466 | 0.00409 – 0.00492 | 3.86 |
| CL_PCT_ | PCT clearance | L/h/70kg | 0.438 | 0.493 | 0.439 0.508 | 3.66 |
| V_PCT_ | PCT volume of distribution | L/70kg | 15 FIX | 15 FIX | | |
| TM_50PCT_ | Maturation half life | weeks | 25.2 | 24.7 | 22.6 – 25.9 | 3.04 |
| Hill_PCT_ | Hill exponent | - | 11.7 | 7.82 | 7.15 – 8.46 | 4.88 |
| Tel_B_ | Elimination half life of birth event | h | 3.58 | 5.67 | 4.99 – 6.20 | 4.63 |
| T_LAG_ | Lag time | h | 19.8 | 12.0 | 9.66 – 14.3 | 9.60 |
| SLOPE_B_ | Linear relationship between birth event concentration and PCT production rate | - | 83.0 | 75.4 | 63.0 – 81.8 | 6.68 |
| Proportional RUV | Proportional error | - | 0.314 | 0.294 | 0.266 – 0.317 | 4.37 |
